# Clonal haematopoiesis and outcomes after acute coronary syndrome: a systematic review and meta-analysis

**DOI:** 10.1093/eschf/xvag191

**Published:** 2026-07-06

**Authors:** Matthew Sadler, Sze Wing Mok, Kaivalya Divekar, Simona Saini, Aamir Shamsi, Ali Malik, Sukruth Kundur, Jamila Salim, Narun Tantichirasakul, Lavan Muraleedharan, Daniela Noakes, Zohal Mohammadi, Tasnim A A Ali, William Chick, Yorissa Padayachee, Sarah Mackie, Mateusz Sokolski, Paul A Scott, Antonio Cannata, Lynn Quek, Daniel I Bromage

**Affiliations:** School of Cardiovascular and Metabolic Medicine & Sciences, King’s College London British Heart Foundation Centre of Excellence, James Black Centre, 125 Coldharbour Lane, London SE5 9NU, UK; Department of Cardiology, King’s College Hospital London, Denmark Hill, Brixton, London SE5 9RS, UK; School of Cardiovascular and Metabolic Medicine & Sciences, King’s College London British Heart Foundation Centre of Excellence, James Black Centre, 125 Coldharbour Lane, London SE5 9NU, UK; Department of Cardiology, King’s College Hospital London, Denmark Hill, Brixton, London SE5 9RS, UK; Peninsula Medical School, Faculty of Health, University of Plymouth, Drake Circus, Plymouth, UK; School of Cardiovascular and Metabolic Medicine & Sciences, King’s College London British Heart Foundation Centre of Excellence, James Black Centre, 125 Coldharbour Lane, London SE5 9NU, UK; Department of Cardiology, King’s College Hospital London, Denmark Hill, Brixton, London SE5 9RS, UK; School of Cardiovascular and Metabolic Medicine & Sciences, King’s College London British Heart Foundation Centre of Excellence, James Black Centre, 125 Coldharbour Lane, London SE5 9NU, UK; School of Cardiovascular and Metabolic Medicine & Sciences, King’s College London British Heart Foundation Centre of Excellence, James Black Centre, 125 Coldharbour Lane, London SE5 9NU, UK; School of Cardiovascular and Metabolic Medicine & Sciences, King’s College London British Heart Foundation Centre of Excellence, James Black Centre, 125 Coldharbour Lane, London SE5 9NU, UK; School of Cardiovascular and Metabolic Medicine & Sciences, King’s College London British Heart Foundation Centre of Excellence, James Black Centre, 125 Coldharbour Lane, London SE5 9NU, UK; School of Cardiovascular and Metabolic Medicine & Sciences, King’s College London British Heart Foundation Centre of Excellence, James Black Centre, 125 Coldharbour Lane, London SE5 9NU, UK; School of Cardiovascular and Metabolic Medicine & Sciences, King’s College London British Heart Foundation Centre of Excellence, James Black Centre, 125 Coldharbour Lane, London SE5 9NU, UK; School of Cardiovascular and Metabolic Medicine & Sciences, King’s College London British Heart Foundation Centre of Excellence, James Black Centre, 125 Coldharbour Lane, London SE5 9NU, UK; School of Cardiovascular and Metabolic Medicine & Sciences, King’s College London British Heart Foundation Centre of Excellence, James Black Centre, 125 Coldharbour Lane, London SE5 9NU, UK; Department of Cardiology, King’s College Hospital London, Denmark Hill, Brixton, London SE5 9RS, UK; Department of Cardiology, Epsom General Hospital, Dorking Road, Epsom, Surrey KT18 7EG, UK; Myeloid Leukaemia Genomics and Biology Group, School of Cancer and Pharmaceutical Sciences, King’s College London, London SE5 8AF, UK; Faculty of Medicine, Institute of Heart Diseases, Wroclaw Medical University, Wroclaw, Poland; Institute of Heart Diseases, University Hospital, Wroclaw, Poland; Department of Cardiology, King’s College Hospital London, Denmark Hill, Brixton, London SE5 9RS, UK; School of Cardiovascular and Metabolic Medicine & Sciences, King’s College London British Heart Foundation Centre of Excellence, James Black Centre, 125 Coldharbour Lane, London SE5 9NU, UK; Department of Cardiology, King’s College Hospital London, Denmark Hill, Brixton, London SE5 9RS, UK; Myeloid Leukaemia Genomics and Biology Group, School of Cancer and Pharmaceutical Sciences, King’s College London, London SE5 8AF, UK; Department of Haematological Medicine, King’s College Hospital NHS Foundation Trust, London, UK; School of Cardiovascular and Metabolic Medicine & Sciences, King’s College London British Heart Foundation Centre of Excellence, James Black Centre, 125 Coldharbour Lane, London SE5 9NU, UK; Department of Cardiology, King’s College Hospital London, Denmark Hill, Brixton, London SE5 9RS, UK

**Keywords:** Clonal haematopoiesis, Clonal haematopoiesis of indeterminate potential, Acute coronary syndrome, Acute coronary syndrome, Outcomes, Inflammation

## Abstract

**Introduction:**

Acute coronary syndrome (ACS) is frequently complicated by left ventricular systolic dysfunction and heart failure. Clonal haematopoiesis of indeterminate potential (CHIP) is characterised by mutations in haematopoietic stem cells that lead to the proliferation of mutant blood cells without overt haematological disease. Whether CHIP is associated with prognosis following ACS remains uncertain.

**Methods:**

We conducted a systematic review and meta-analysis of observational studies that compared outcomes after ACS in patients with and without CHIP. The primary outcome was all-cause mortality. Exploratory subgroup analyses explored mutation class and study-level comorbidities.

**Results:**

Eight studies comprising diverse ACS populations, from low-risk ACS to cardiogenic shock, were included. In the primary analysis, CHIP was associated with higher all-cause mortality [pooled hazard ratio 1.61, 95% confidence interval 1.36–1.92], with no detected statistical heterogeneity (*I*^2^ = 0%). Mutation-specific analyses were underpowered but suggested a more consistent association between Ten-eleven translocation 2 mutations and adverse events. Exploratory study-level analyses suggested that differences in smoking prevalence between CHIP carriers and non-carriers may contribute to variability in association strength, but no formal interaction testing was possible.

**Conclusion:**

Clonal haematopoiesis of indeterminate potential is associated with increased mortality following ACS across a broad range of clinical settings, suggesting it is a marker of adverse post-ACS risk. These findings highlight the need for adequately powered prospective studies with standardized CHIP ascertainment to determine whether CHIP represents a feasible biomarker or modifiable therapeutic target.

## Graphical Abstract

**Graphical Abstract:**
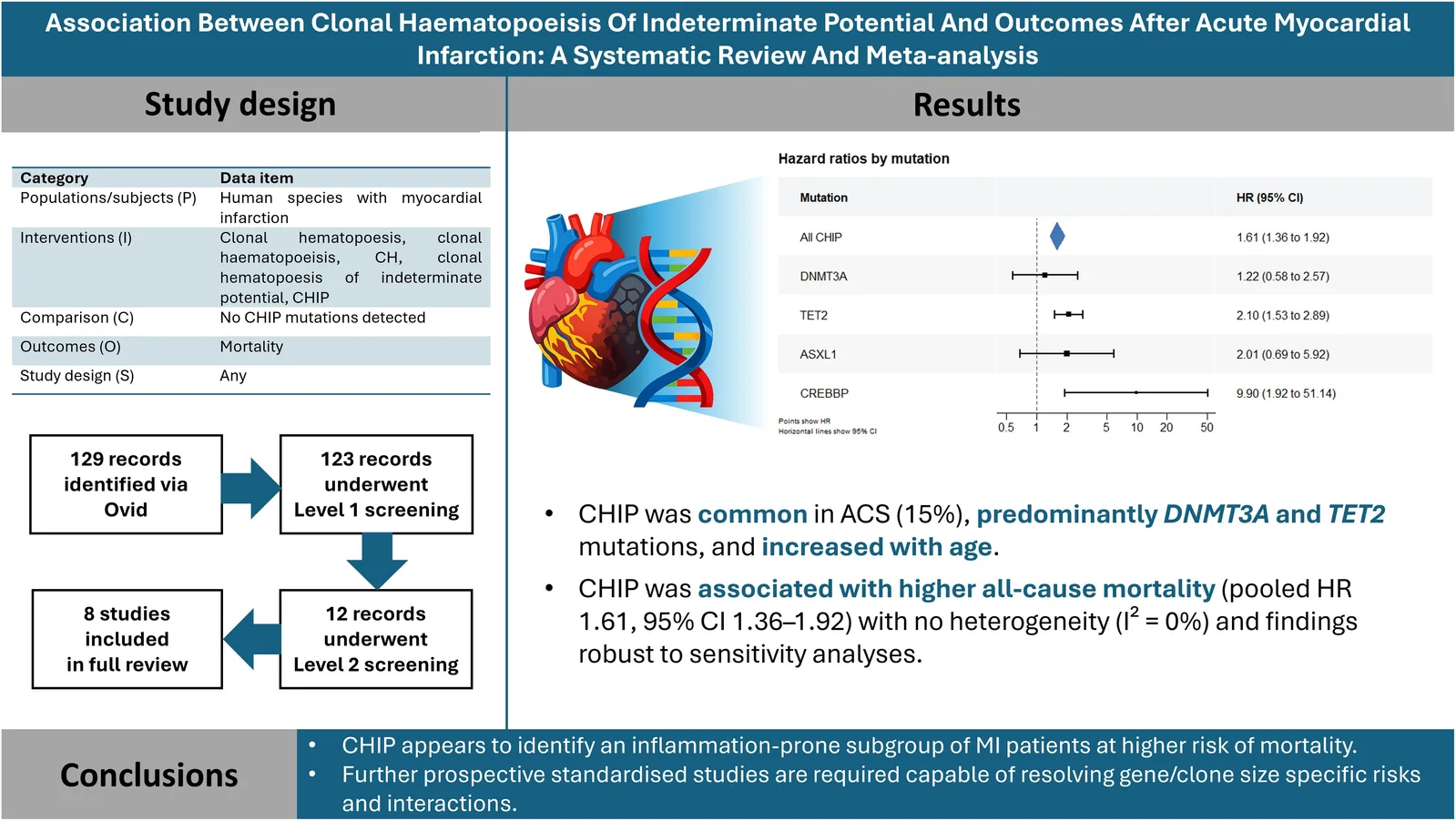

## Introduction

The inflammatory response to acute coronary syndrome (ACS) is essential for debris clearance and myocardial repair.^1^ However, excessive or prolonged inflammation has consistently been associated with maladaptive ventricular remodelling and adverse outcomes.^2,3^ Achieving an appropriate balance is therefore critical: persistent inflammation exacerbates myocardial injury, whereas excessive suppression may impair scar formation and healing.^4^

Clonal haematopoiesis (CH) is defined by the acquisition of somatic mutations in haematopoietic stem and progenitor cells (HSPCs) that confer a fitness advantage, leading to expanded mutant blood cell clones in the absence of overt haematological disease. By convention, when the largest clone exceeds a variant allele frequency (VAF) of 2%, it is termed CH of indeterminate potential (CHIP). The most common CHIP driver genes include DNA methyltransferase 3A (*DNMT3A*), ten-eleven translocation 2 (*TET2*), and additional sex combs-like 1 (*ASXL1*).^5^

Clonal haematopoiesis of indeterminate potential is associated with predominantly pro-inflammatory responses, incident atherosclerotic cardiovascular disease (CVD) and worse outcomes in established disease,^6^ with experimental evidence supporting a causal association with atherogenesis.^7^ Acute coronary syndrome represents an acute inflammatory stress in which the dysregulated innate immune responses associated with CHIP may contribute to maladaptive remodelling, left ventricular systolic dysfunction, and heart failure (HF).^8^ While several observational studies have reported associations between CHIP and outcomes following ACS,^9–14^ these studies differ substantially in methodology. As a result, CHIP-associated risk after ACS remains uncertain, particularly with respect to mutation class, clonal burden, and interaction with comorbidities. We therefore undertook a systematic review and meta-analysis to provide a comprehensive estimate of the association between CHIP and outcomes after ACS.

## Methods

This systematic review and meta-analysis were conducted in accordance with Preferred Reporting Items for Systematic reviews and Meta-Analyses (PRISMA) guidelines.^15^ The review protocol was not prospectively registered. All eligibility criteria, outcomes, and analytic approaches were specified a priori.

### Search strategy and eligibility criteria

Eligibility criteria were defined using the Population, Intervention, Comparison, Outcomes, and Study (PICOS) approach. The PICOS statement was developed by two independent reviewers (M.S. and D.B.) using published guidelines and peer reviewed by members of the King’s College London NHS Trust Heart Failure Unit Research Group.

We included human studies that evaluated the association of CHIP, per each study’s definition (typically VAF ≥2%) and regardless of specific driver mutation, with all-cause mortality following ACS, compared to patients without CHIP, irrespective of study design (*Table 1*).

**Table 1:** PICOS (population, intervention, comparison, outcomes, and study) criteria for formulating eligibility criteria for the study

Category	Data item
Populations/subjects (P)	Human patients with acute coronary syndrome
Interventions (I)	Clonal hematopoesis, clonal haematopoeisis, CH, clonal hematopoiesis of indeterminate potential, CHIP
Comparison (C)	No CHIP mutations detected
Outcomes (O)	Mortality
Study design (S)	Any

CH, clonal haematopoiesis; CHIP, clonal haematopoiesis of indeterminate potential

A systematic review of Medline (1946 to 26 September 2025) and Embase (1974 to 26 September 2025) was conducted by M.S. and C.M. The search strategy combined controlled vocabulary and free-text terms relating to ACS and CHIP, based on the PICOS criteria and adapted to each database (Supplementary material and Supplementary Table S1). Searches were limited to reports available in English due to limited resources for translation. Review articles, abstract articles, unpublished material and ongoing studies were excluded. Duplicates were removed using Ovid (Wolters Kluwer, NL).

### Study selection

Retrieved records were screened for eligibility using the title and abstract (Level 1 screening), followed by the full text (Level 2 screening). Screening was performed independently by two reviewers (C.M. and M.S.) using predefined eligibility fields. Discrepancies were resolved by consensus after re-examining the relevant record.

### Data extraction and outcomes

Two reviewers (C.M. and M.S.) independently extracted study variables using a piloted template, resolving discrepancies by consensus. For each study, we recorded the clinical context (including ACS phenotype), CHIP ascertainment (mutant gene(s) and VAF threshold), timing of blood sampling relative to the index event, outcome definitions, follow-up, and effect estimates. Where multiple models were reported, multivariable estimates with the most comprehensive covariate adjustment were selected. The primary outcome was all-cause mortality.

### Risk of bias

Risk of bias was assessed using the ROBINS-E tool based on two independent reviewers’ domain-level judgements (S.S. and A.M.),^16^ mapped to categories (low risk, some concerns, high risk) and resolved by consensus, with any unresolved disagreements conservatively assigned the more severe judgement. Overall study-level risk of bias was assigned after consensus review of the domain-level judgements, using a conservative approach in which concerns in any key domain informed the overall classification.

### Quantitative synthesis

Between-study heterogeneity was anticipated and therefore random-effects models with restricted maximum likelihood were used. For each study, we extracted the most fully adjusted time-to-event effect estimate for all-cause mortality and used the hazard ratio (HR) as the principal effect measure. Covariates included in the adjusted models varied between studies and are summarized in Supplementary Table S2. Where several adjusted models were available, we selected the model judged most clinically comprehensive, prioritizing age, sex, cardiovascular risk factors, infarct characteristics, and treatment variables where reported. Because follow-up durations varied across studies, HRs were synthesized on the log scale (with corresponding standard errors) without converting to a common follow-up time. Where a study did not report an HR, an approximate unadjusted HR was reconstructed from published Kaplan–Meier data using extracted time-point event counts and numbers at risk. The resulting HR and 95% confidence interval were then converted to log(HR) and standard error using the generic inverse-variance method. Standard errors were derived from CI width using standard methods. Where studies reported group-specific estimates, we avoided double-counting by combining group-specific log(HR) using inverse-variance weighting.

Between-study heterogeneity was assessed using χ^2^, τ^2^, *I*^2^, and 95% prediction intervals; heterogeneity was considered substantial when *I*^2^ exceeded 75%.^17^

Continuous variables were summarized as median (interquartile range) or mean (standard deviation) and categorical variables as counts (percentages). Statistical significance was set at two-sided α = .05. Analyses were performed in R (version 4.4.1).

### Assessment and handling of potentially overlapping cohorts

Because overlap between cohorts could not be definitively excluded, potentially overlapping studies were retained in the primary analyses to avoid subjective exclusion of relevant data. Potential overlap between publications was addressed using structured identifiers, including institution, recruitment dates, and methodology. Within each unique cohort, we retained the most comprehensive adjusted estimate and mutation-specific estimates. Where the highest-priority report did not provide the required mutation-level information, an alternative report from the same cohort was used.

### Subgroups and sensitivity analyses

Prespecified subgroup analyses explored mutation classes and study-level covariates. Study-level covariates were extracted from baseline characteristics tables. Where baseline data were reported separately by CHIP status, group-level differences were calculated and presented alongside effect estimates. No formal adjustment for multiple testing was applied, reflecting the exploratory nature of these analyses and limited statistical power. Pre-specified sensitivity analyses comprised (i) an adjusted-only dataset, (ii) a dataset excluding potentially overlapping cohorts, and (c) leave-one-out and influence diagnostics.

### Publication bias and small-study effects

Small-study effects were assessed when at least three studies were available by visual inspection of contour-enhanced funnel plots and formal testing using Egger’s regression and Begg’s rank-correlation tests.

## Results

### Study selection

The systematic search identified 129 studies. After removing duplicates and screening for eligibility, eight studies were included in our review (study selection flowchart in *Figure 1*).

**Figure 1:**
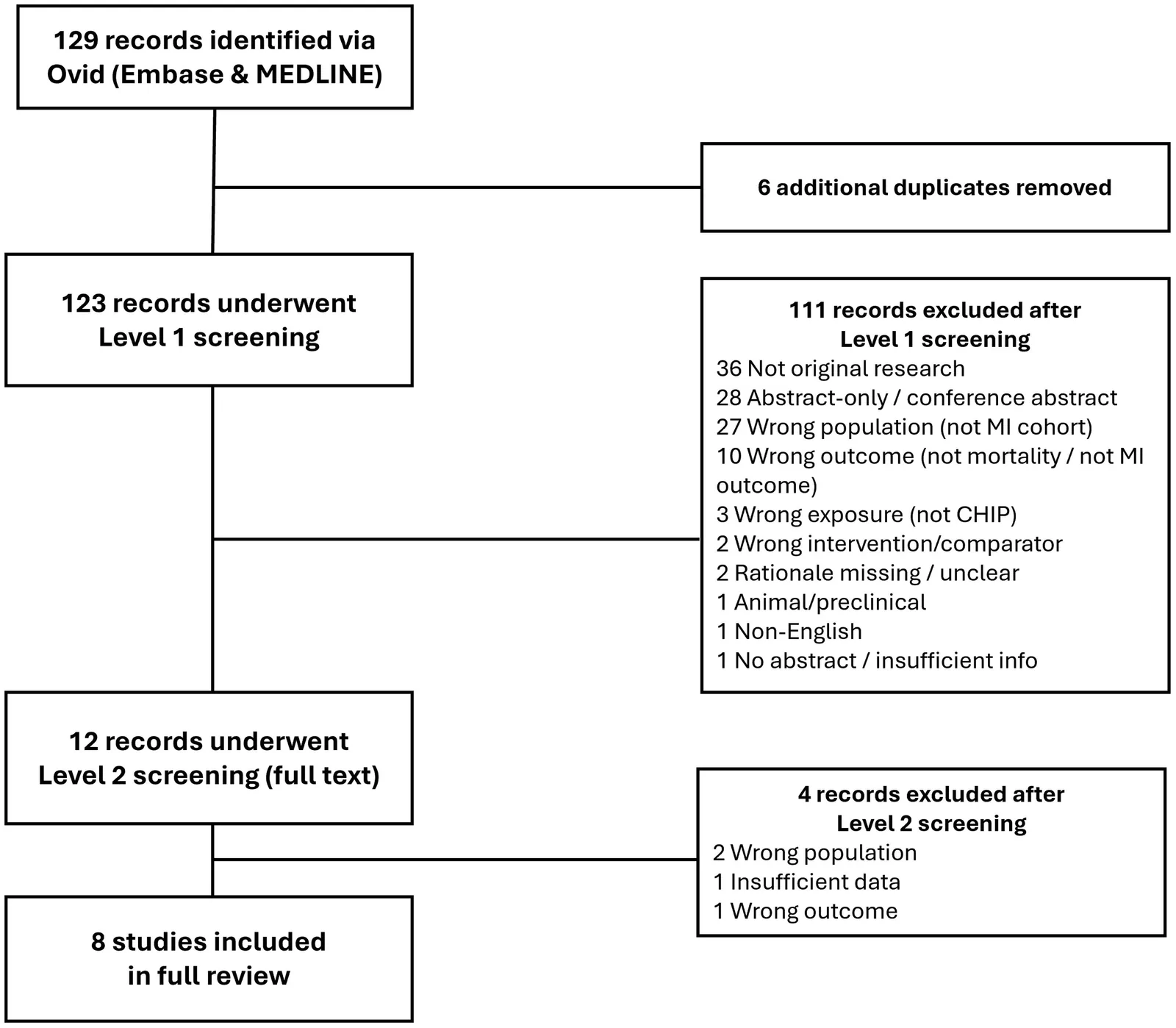
Study selection. PRISMA flow diagram showing identification, screening, and inclusion of studies assessing clonal haematopoiesis of indeterminate potential and outcomes following acute coronary syndrome. Eight studies were included in the final synthesis

### Risk of bias

No study was judged to be at high risk of bias in any domain (*Figure 2*). Overall, four studies were classified as having some concerns, while four were judged to be at low risk of bias. The most common source of concern related to bias due to confounding (Domain 1), reflecting incomplete adjustment for key covariates.

**Figure 2:**
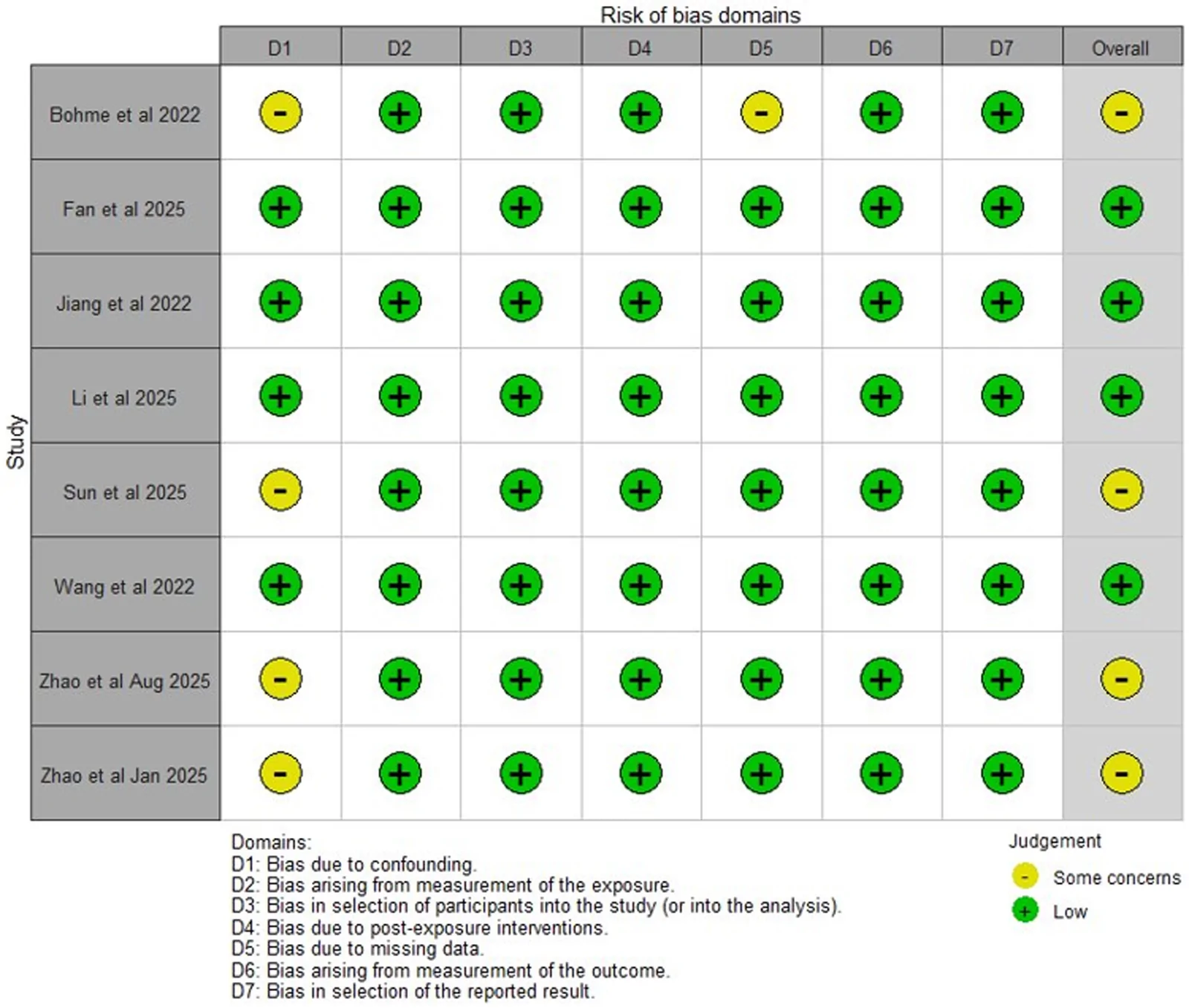
Risk of bias assessment. Summary of domain-level risk of bias across included observational studies, assessed using ROBINS-E. Overall judgements reflect consensus review of domain-level assessments

### Publication bias and small-study effects

Visual inspection of contour-enhanced funnel plots did not identify marked small-study effects (*Figure 3*). We performed formal testing for asymmetry using Egger’s regression and Begg’s rank-correlation. Neither test reached statistical significance (Egger *P* = .142; Begg *P* = .083), although their power was limited by the modest number of contributing studies. Accordingly, these analyses should be interpreted cautiously and do not exclude publication bias.

**Figure 3:**
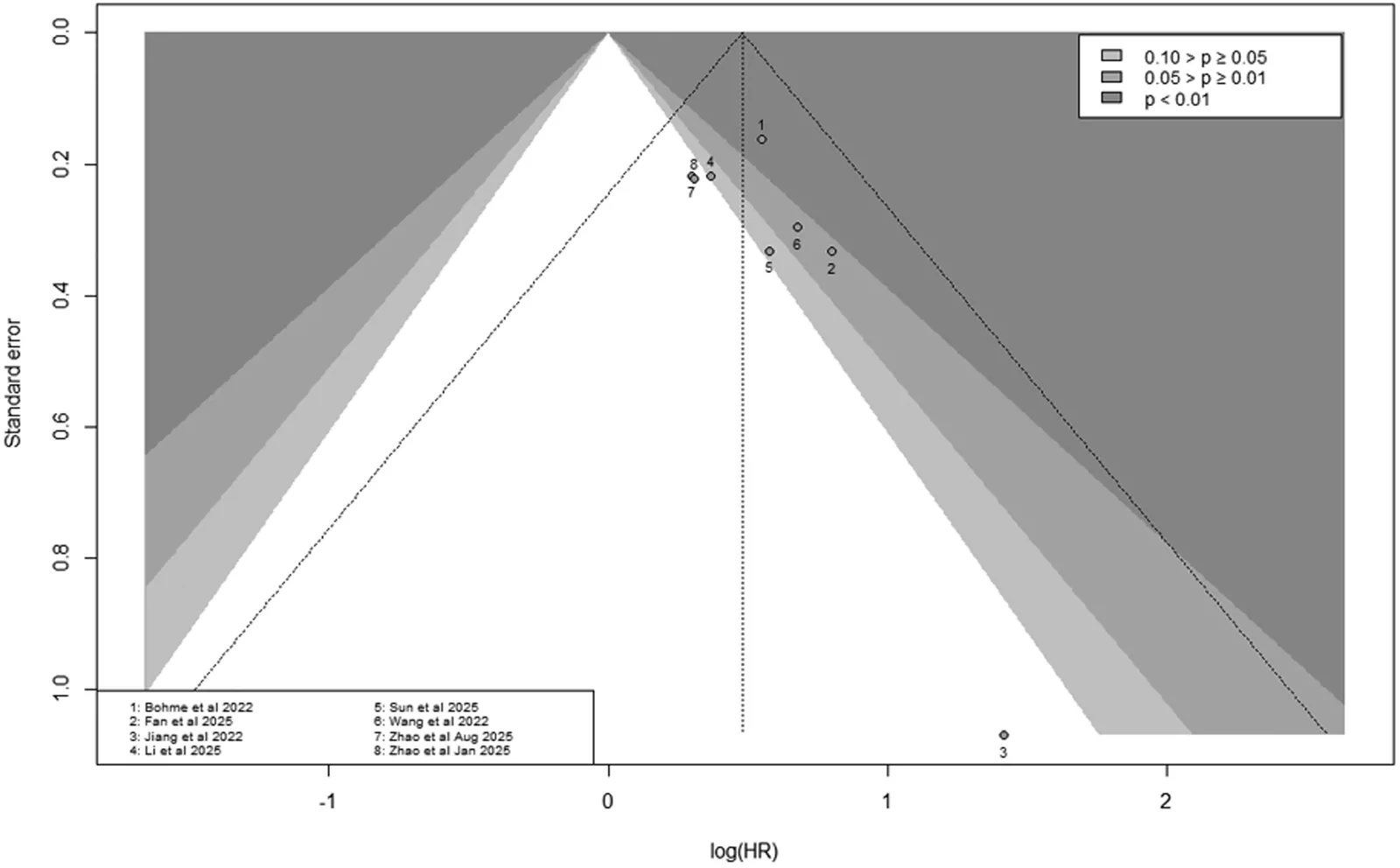
Funnel plot. Contour-enhanced funnel plot assessing small-study effects for the primary all-cause mortality analysis. Interpretation is limited by the small number of included studies

### Qualitative review of included studies

#### Study design and patient characteristics

Clinical context and baseline risk varied considerably between studies, which reflect a continuum of clinical severity, including low-risk ACS without cardiovascular risk factors (CVRFs), cardiogenic shock with very high short-term mortality, and general myocardial infarction (MI) cohorts (study characteristics summarized in *Table 2*). For example, Jiang *et al.* analysed 179 ACS patients, including ST-segment elevation MI (STEMI, 43%), non-STEMI (19%), and unstable angina (38%).^10^ Stringent eligibility criteria excluded traditional CVRFs, prior CVD, lipid-lowering therapy, myelodysplastic syndromes (MDS), and active malignancy (eligibility criteria of included studies summarized in *Table 3*). Given the stringent inclusion criteria, this cohort had the lowest overall event rate. In contrast, Böhme *et al.* reported a sub-study of the CULPRIT-SHOCK trial, which randomized patients with MI (∼60% STEMI) complicated by cardiogenic shock and multivessel disease to culprit-lesion-only or multivessel percutaneous coronary intervention (PCI).^11,20^ Event rates in this cohort were extremely high, consistent with a very severe MI population (endpoints of included studies summarized in *Table 4*).

**Table 2:** Study characteristics

Characteristic	Overall (pooled)	Böhme et al.^11^	Jiang et al.^10^	Wang et al.^9^	Fan et al.^14^	Li et al.^13^	Sun et al.^18^	Zhao et al.^19^	Zhao et al.^12^
**Country**		European multicentre	China	China	Taiwan	China	China	China	China
**Design and population**		RCT sub-study (CULPRIT-SHOCK): AMI with cardiogenic shock, multivessel disease	Prospective ACS cohort (STEMI/NSTEMI/UA) with stringent CVRF exclusions	Prospective STEMI cohort; random sample for CHIP	Prospective STEMI cohort (controls also enrolled)	Prospective AMI programme with intracoronary imaging subset	Prospective STEMI cohort; patients with OCT included	Prospective AMI cohort (STEMI and NSTEMI) with focus on atherogenic index of plasma (AIP)	Prospective AMI (STEMI and NSTEMI) programme with T2DM focus and MR analyses
**Recruitment period**		2013–2017	2018–2019	2017–2019	From 2011	2017–2020	2017–2019	2017–2020	2017–2020
**N analysed for CHIP**	6041	446	179	485	101	1403	628	1396	1403
**Sampling window**		Peri-procedural/randomisation	Day after admission	During index revascularisation	Day 28 post-STEMI (subset immediate post-PCI)	Baseline (admission/PCI)	During index revascularisation	Baseline (pre-angiography)	Baseline (pre-angiography)
**Reperfusion**		Culprit-only vs multivessel PCI	PCI (dominant)	Thrombolysis or PCI	PCI (routine care)	PCI (routine care)	PCI (dominant)	PCI (dominant)	PCI (dominant)
**Follow-up duration**		30 days	Up to 5 years	Median 3 years	Median ∼10 years	Scheduled ≥12 months, then annual	Median 2.4 years	Median 3.99 years	Median ∼4 years

Baseline characteristics	CHIP	Non-CHIP	CHIP	Non-CHIP	CHIP	Non-CHIP	CHIP	Non-CHIP	CHIP	Non-CHIP	CHIP	Non-CHIP	CHIP	Non-CHIP	CHIP	Non-CHIP	CHIP	Non-CHIP
**N**	905	5136	129	317	36	143	60	425	38	63	157	1246	77	551	203	1193	205	1198
**Age, mean (SD)**	66.1 (12.3)	60.0 (12.3)	73.0 (12.6)	67.0 (12.6)	70.0 (10.6)	66.4 (9.0)	66.5 (10.7)	61.1 (12.0)	65.2 (10.9)	58.6 (11.1)	65.8 (12.5)	59.8 (12.1)	62.5 (9.8)	55.6 (11.7)	64.5 (12.2)	59.7 (12.2)	64.5 (12.2)	59.7 (12.2)
**BMI, mean (SD)**	25.5 (3.8)	25.6 (4.3)	27.2 (3.6)	26.4 (3.5)	24.4 (3.3)	24.6 (3.6)	24.2 (3.0)	24.6 (3.3)	25.4 (3.4)	28.9 (16.4)	25.5 (3.9)	25.9 (3.6)	24.1 (4.1)	25.1 (3.3)				
**Female, %**	26.7	20.7	27.1	23.3	52.8	36.4	41.7	25.4	10.8	10.9	23.6	18.5	41.6	26.7	21.7	18.5	22.0	18.6
**Smoker, %**	58.1	65.9	19.4	27.8			50.0	52.9	56.8	54.7	67.5	72.4	53.2	57.9	69.0	72.0	68.8	71.9
**Hypertension, %**	60.9	60.2	58.9	65.0			53.3	45.2	56.8	53.1	64.3	64.2	48.1	43.2	64.0	64.4	64.4	64.1

ACS, acute coronary syndrome; AMI, acute myocardial infarction; CHIP, clonal haematopoiesis of indeterminate potential; CVRF, cardiovascular risk factors; MR, Mendelian randomization; PCI, percutaneous coronary intervention; STEMI, ST-elevation MI; UA, unstable angina. OCT, optical coherence tomography. AMI, acute myocardial infarction.

**Table 3:** Eligibility criteria and exclusion criteria of each study

Study	Key eligibility	Key exclusions
Jiang et al.^10^	ACS undergoing PCI; baseline genotyping	Traditional CVRFs (smoking, hypertension, diabetes, dyslipidaemia); prior CVD (MI, stroke, PAD, prior PCI/CABG); active malignancy/suspected MDS; inadequate DNA; QC failures
Böhme et al.^11^	AMI with cardiogenic shock; multivessel CAD; identifiable culprit; planned early PCI	Resuscitation >30 min; no intrinsic heart action; fixed dilated pupils; indication for urgent CABG; single-vessel CAD; mechanical cause of shock; shock onset >12 h; age >90 y; non-cardiogenic shock; massive PE; severe renal insufficiency; severe comorbidity (life expectancy <6 months); no stored sample
Wang et al.^9^	STEMI; reperfusion by thrombolysis or PCI	Haematological malignancy (pancytopenia of unknown aetiology); NSTEMI; no intervention; no stent after reperfusion; not selected in random sampling; insufficient sample; inadequate DNA; in-hospital death (pre-sampling)
Li et al.^13^	AMI; baseline genotyping; imaging subset pre-intervention OCT	Programme-level standard AMI exclusions; imaging subset excluded cardiogenic shock; other specifics NR
Zhao et al.^a12^	AMI; PCI; baseline genotyping; prespecified T2DM subgroups	History of malignancy and severe comorbidity (per protocol); other specifics NR
Fan et al.^14^	STEMI survivors to day 28; controls without CVD	Prior haematological disorders; inadequate sample; controls: any CVD
Zhao et al.^b19^	AMI; PCI; baseline genotyping; lipid measurements;	Malignancy (active or history); moderate-severe valvulopathy; stage 4–5 CKD, moderate hepatic dysfunction
Sun et al.^18^	STEMI; baseline genotyping; pre-intervention OCT	Poor quality OCT images, in-stent restenosis, extensive thrombosis, balloon pre-dilatation prior to OCT.

CABG, coronary artery bypass graft; CAD, coronary artery disease; CHIP, clonal haematopoiesis of indeterminate potential; CVD, cardiovascular disease; MDS, myelodysplastic syndromes; NR, not reported; OCT, optical coherence tomography; PAD, peripheral arterial disease; PE, pulmonary embolism; QC, quality control. AMI, acute myocardial infarction; CKD, chronic kidney disease. ^a^Published in January 2025; ^b^Published in August 2025.

**Table 4:** Primary and secondary endpoints of each study

Study	Primary endpoint(s)	Key secondary endpoint(s)	Endpoint notes
Jiang et al.^10^	All-cause mortality and MACCE (death, non-fatal MI, non-fatal stroke, UA with unplanned revascularization)	Component events, where reported	Adjudicated; low absolute event rate due to stringent exclusions
Böhme et al.^11^	30-day composite: all-cause death or renal replacement therapy	All-cause death; recurrent MI; other trial-defined events	Shock biology; very high early event rate; no HF-specific endpoint in primary composite
Wang et al.^9^	MACE: all-cause death, recurrent MI, stroke, HF hospitalization	Component-wise analyses	Median 3-year follow-up; cytokine sub-studies (IL-1β/IL-6)
Li et al.^13^	Composite clinical events: all-cause death, MI, stroke, ischaemia-driven revascularization	Imaging endpoints: thin-cap fibroatheroma, lipid/necrotic core metrics; gene/clone-size associations	Imaging subset pre-PCI; links genotype→plaque vulnerability
Zhao et al.^12^	All-cause mortality	MACE components where reported; gene × T2DM interactions; MR outcomes	Diabetes-stratified analyses; MR supports CHIP↔T2DM links
Fan et al.^14^	MACE: all-cause death, recurrent MI, stroke, revascularization, HF hospitalization	Component-wise where available	Day 28 sampling; very long-term horizon; potential survivor bias for very early events
Zhao et al.^19^	All-cause mortality	MACE, defined as all-cause death, cardiac death, recurrent MI, recurrent heart failure, ischaemic stroke, unplanned repeat revascularization	Follow-up was protocolized: 1 month in-person, then 6 and 12 months, then annual thereafter; phone follow-up if unable to attend. Events were adjudicated by an independent clinical event committee, with monthly data updates to the research team
Sun et al.^18^	MACE: all-cause death, nonfatal MI or stroke, ischaemia-driven revascularization.	All-cause mortality	Events adjudicated by an independent clinical committee

HIP, CHIP, clonal haematopoiesis of indeterminate potential; HF, heart failure; MI, myocardial infarction; T2DM, type 2 diabetes mellitus.

Fan *et al.* reported a prospective STEMI cohort (*n* = 101 STEMI, 706 controls without CVD),^14^ Unlike the other studies, which performed baseline peripheral blood sampling at or shortly after the index admission/PCI, sampling was at 28 days post-STEMI.

Li *et al.* and Zhao *et al.* reported multiple complementary analyses from the single-centre Fuwai/CRGAM acute STEMI programme, together comprising the largest contemporary PCI-treated STEMI cohort in this review. Li *et al.* enrolled 1403 consecutive STEMI patients, with comprehensive clinical follow-up and, in a subset (*n* = 355) undergoing pre-interventional optical coherence tomography, plaque morphology was characterized alongside intravascular ultrasound in routine care. Zhao *et al.* studied 1403 consecutive PCI-treated STEMI patients from the same programme with a prespecified focus on the interaction between CHIP and type 2 diabetes mellitus (T2DM).^12^ Zhao *et al.* reported a second analysis from the same cohort with extended follow-up and refined multivariable adjustment.^19^ Given the overlapping enrolment periods, single-centre setting, and shared recruitment pipeline, substantial participant overlap between the Li and Zhao cohorts is likely.^12,13,19^ Accordingly, Li’s more comprehensive dataset was prioritized for quantitative pooling in a sensitivity analysis, while Zhao’s analyses were used to inform subgroup analysis in patients with diabetes.

#### CHIP ascertainment and variant calling

Across studies, CHIP was assessed in peripheral blood using next-generation sequencing with prespecified VAF thresholds. Most cohorts used VAF ≥2% as the primary definition and, where possible, reported additional analyses for low-VAF clones (1–2%). However, important methodological differences in panel breadth, sequencing depth, and analytic grouping remain, with implications for both prevalence estimates and effect sizes.

Whole-exome approaches applied at ∼200× depth classified participants according to any-CHIP and grouped common driver subsets,^10,14^ but the modest depth reduces the sensitivity for small clones and clone-size analyses. By contrast, targeted panels sequencing a small number of canonical genes at very high coverage enabled clearer separation of CHIP-negative, low-VAF, and CHIP-positive groups, with more reliable assessment of gene-specific and clone-size analyses.^11^ Broader targeted panels, including high-depth approaches without unique molecular identifiers (UMIs) and, in several studies, UMI-based error-corrected sequencing at ≥1000× to >10 000× depth, expanded coverage and allowed analyses across multiple VAF cut-offs, including <1%.^9,13,18,19^ Therefore, we identified substantial heterogeneity in driver coverage and groupings and VAF thresholds (>0.5%, ≥1%, ≥2%). Accordingly, for quantitative synthesis we harmonized the primary exposure to VAF ≥2%, which was available across all studies. However, this harmonization does not remove differences in sequencing depth, driver-gene coverage, error-correction methods, or variant-calling pipelines, which may influence both CHIP prevalence and study-level effect estimates.

#### Study findings and interpretation

Despite methodological variation, all included studies report that CHIP is common among ACS patients and associated with worse outcomes. Prevalence rose steeply with age in every cohort, and *DNMT3A* and *TET2* were consistently the most prevalent drivers. Mechanistically, CHIP was linked to a pro-inflammatory milieu (notably higher IL-1β and IL-6) and, in imaging data, features of plaque vulnerability.

In Jiang’s low-risk ACS cohort, 36/179 patients carried CHIP mutations (predominantly *DNMT3A*).^10^ Age-adjusted Cox models associated *DNMT3A*/*TET2*/*ASXL1* with higher event risk, and IL-6 was the cytokine most consistently elevated across *DNMT3A*, *TET2* and *ASXL1* carriers. Similarly, in Böhme’s cardiogenic shock cohort, 129/446 carried CHIP at VAF ≥2%, most commonly *DNMT3A*.^11^ Clonal haematopoiesis of indeterminate potential status was associated with higher odds of the primary composite endpoint (30 day all-cause death or renal replacement therapy [RRT]), after adjustment for age, sex, PCI strategy, and the CLIP biomarker score (cystatin C, lactate, IL-6, and N-terminal pro-B-type natriuretic peptide [NT-proBNP]). The association with 30-day mortality alone was directionally consistent but was not significant in fully adjusted models. Notably, low-VAF clones (1–2%) were not associated with the composite endpoint in adjusted models; however, analyses treating VAF as a continuous variable were consistent with an association between worse outcomes and higher clone size.

In Wang’s STEMI study, *DNMT3A*/*TET2* carriers at VAF ≥2% had higher all-cause mortality and MACE over 3 years, compared to individuals without CHIP, and exhibited elevation of IL-1β and IL-6, whereas TNF-α and IFN-γ did not differ.^9^ Li’s imaging-enriched cohort linked any-CHIP (≥2% VAF) to thin-cap fibroatheroma and larger lipid cores, with higher event rates over follow-up.^13^ Associations were most evident for *TET2*/*ASXL1*. Zhao’s analysis similarly demonstrated an association with adverse outcomes in CHIP carriers, with stronger associations in T2DM and supportive Mendelian randomization, suggesting metabolic–inflammatory interaction.^12^ A second Zhao *et al*. analysis with extended follow-up yielded directionally consistent estimates.^19^ Likewise, Fan’s long-term registry demonstrated a durable association with excess risk over a median of ∼10 years, with mortality and MACE persistently higher in CHIP carriers.^14^

These findings collectively support the biological plausibility of an association between CHIP and adverse post-ACS trajectories, involving myeloid inflammation, plaque vulnerability, and amplification by chronic inflammatory stressors such as T2DM, with a clone-size gradient where VAF strata were reported.

### Quantitative analysis

#### All-cause mortality

In our meta-analysis, CHIP was associated with higher all-cause mortality (pooled HR 1.61, 95% CI 1.36–1.92), with no detected statistical heterogeneity (*I*^2^ = 0%, τ^2^ = 0; Cochran Q *P* = .786; *Figure 4*). Across individual studies, point estimates were directionally concordant. However, given the small number of included studies and clear differences in clinical setting, sampling timing, sequencing methodology, and outcome definitions, this should not be interpreted as evidence of clinical or methodological homogeneity.

**Figure 4:**
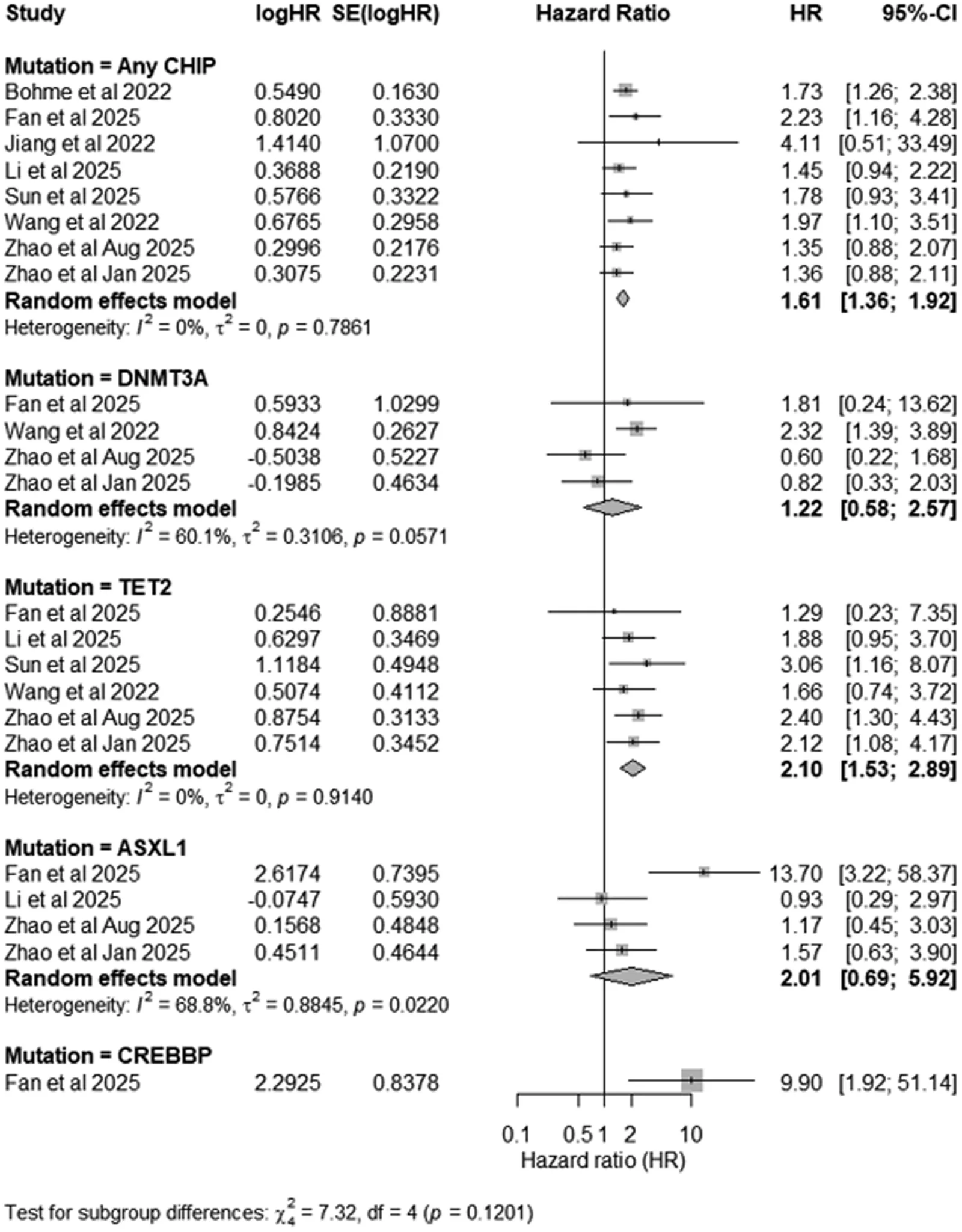
Clonal haematopoiesis of indeterminate potential (CHIP) and all-cause mortality after acute coronary syndrome. Forest plot of adjusted hazard ratios for the association between CHIP and all-cause mortality. Gene-specific estimates are shown for *DNMT3A*, *TET2*, and *ASXL1* CHIP mutations where available

#### Subgroup and sensitivity analyses

Gene-stratified analyses were broadly consistent. *TET2*-CHIP was associated with increased mortality (HR 2.10, 95% CI 1.53–2.89; *I*^2^ = 0%; *Figure 4*), whereas *DNMT3A*- and *ASXL1*-CHIP showed more heterogeneous associations (HR 1.22, 95% CI 0.58–2.57; *I*^2^ = 60.1% and HR 2.01, 95% CI 0.69–5.92; *I*^2^ = 68.8%, respectively), consistent with lower event rates (*Figure 4*). A global test for subgroup differences across mutation strata was not statistically significant (χ^2^ = 7.32, df = 4, *P* = .120), consistent with limited power for between-gene comparisons.

Clonal haematopoiesis of indeterminate potential carriers were consistently older and differed modestly in baseline cardiovascular risk profiles, including sex distribution, smoking status, body mass index, and prevalence of hypertension (*Table 1*). Across most studies, smoking prevalence was lower among CHIP carriers than non-carriers, whereas differences in BMI and hypertension were more variable or modest (*Figure 5*). Populations in which smoking was more prevalent were additionally observed to have more modest associations between CHIP and all-cause mortality.

**Figure 5:**
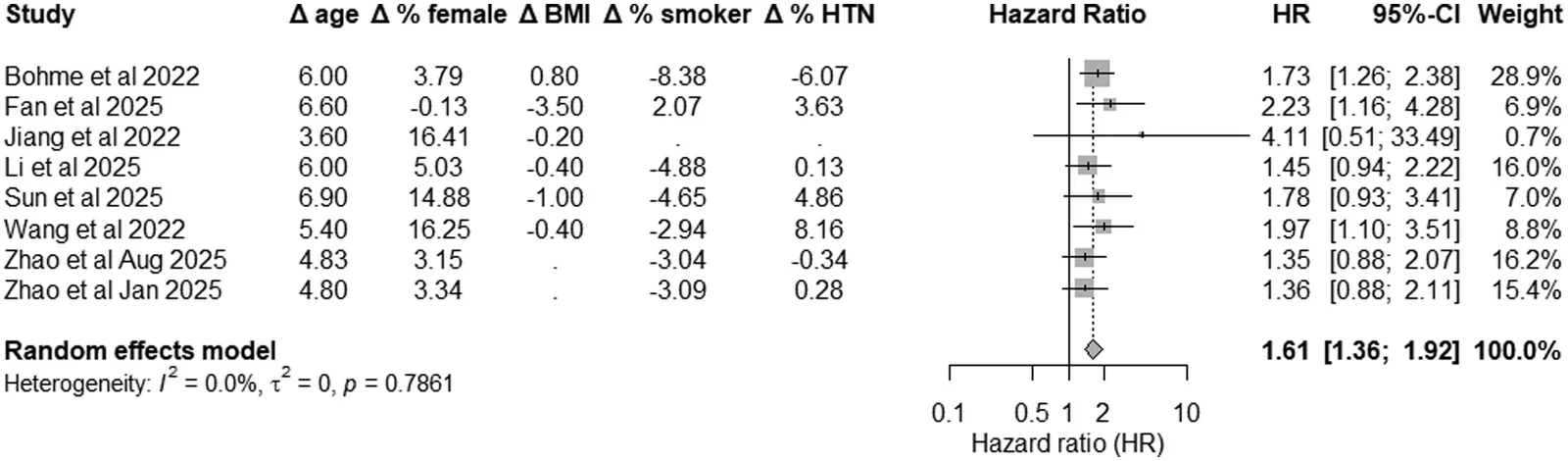
Exploratory study-level covariate analyses. Study-level differences in baseline clinical characteristics between clonal haematopoiesis of indeterminate potential (CHIP) carriers and non-carriers are shown alongside hazard ratios for all-cause mortality. These analyses are exploratory and do not represent individual-level interaction testing

To address potential duplication, we identified potentially overlapping cohorts. This included three analyses from the Fuwai/CRGAM programme,^12,13,19^ and a further two from Harbin Medical University,^9,18^ resulting in a slightly larger pooled estimate (HR 1.75, 95% CI 1.40–2.18; I^2^ = 0%, τ^2^ = 0; *P* = .720; Supplementary Figure S1). Restricting to adjusted estimates only attenuated the pooled association modestly but remained statistically significant (HR 1.55, 95% CI 1.27–1.91; *I^2^* = 0%, τ^2^ = 0; *P* = .721; Supplementary Figure S2). Leave-one-out influence diagnostics for the primary analysis showed minimal dependence on any single study, with pooled estimates ranging from HR 1.57 to 1.67 (all *P* < .0001, I^2^ = 0%; Supplementary Figure S3). Leave-one-out analyses performed for sensitivity analyses and mutation-specific subgroups did not identify single-study dependence but were limited by small numbers of contributing studies (Supplementary Figures S4 and S5).

## Discussion

Large-scale population studies first established CHIP as an age-associated state with increased risk of haematological malignancy and all-cause mortality.^21,22^ Building on this, a pivotal case-control meta-analysis demonstrated that CHIP nearly doubled the risk of coronary heart disease and increased the risk of early-onset MI four-fold, with *DNMT3A*, *TET2*, and *ASXL1* the most frequent drivers.^8^ These epidemiological signals were supported by experimental work showing that *Tet2*-deficient haematopoiesis accelerates atherosclerosis via heightened IL-1β signalling.^23,24^ Together, evidence to date provides a plausible causal framework in which CHIP augments innate immune activation to worsen ischaemic heart disease incidence.

Several studies have extended the association from incident atherosclerotic CVD to outcomes after ACS. Pooling data from observational cohorts, we found that CHIP was associated with a ∼60% increase in all-cause mortality, with directionally consistent associations across low-risk ACS, unselected STEMI, and cardiogenic shock populations, and no detected statistical heterogeneity. This statistical consistency should be interpreted alongside substantial clinical and methodological heterogeneity between the included cohorts. In a low-risk ACS cohort stringently excluding CVRFs, CHIP carriers nonetheless experienced more major adverse cardiac or cerebral events (MACCE), implying that clonal inflammation confers residual risk independent of conventional pathways.^10^ Additionally, in cardiogenic shock, an extreme inflammatory milieu, CHIP remained associated with substantially higher 30 day mortality and renal failure.^11^ Across STEMI cohorts, excess risk appeared durable, arguing against CHIP as a short-lived epiphenomenon and supporting its potential value as a marker of longer-term adverse risk.^9,14^ Mechanistically, the Fuwai/CRGAM imaging sub-study linked CHIP to vulnerable plaque morphology, providing an association between genotype, lesion biology, and clinical events after MI.^13^

A second contribution is gene- and clone-size specificity. Across datasets, *DNMT3A* and *TET2* dominate, although mutation-specific hazard estimates were overlapping. Although *DNMT3A* was the most prevalent driver across studies, reflecting its prevalence in population studies,^8^  *DNMT3A* mutations are also heterogeneous, especially missense mutations, and this heterogeneity may attenuate the association between *DNMT3A* CHIP and outcomes. Mutation-specific analyses suggested a more consistent association between *TET2* mutations and adverse events but were underpowered to draw firm conclusions. For now, CHIP should be interpreted as a composite exposure in this context, until further evidence for gene-specific effects becomes available. Most studies used a VAF threshold of 2%. The shock cohort demonstrated that categorical low-VAF (1–2%) clones were not consistently associated with adverse outcomes, whereas analyses treating VAF continuously supported increasing risk with greater clonal burden.^11^

Multiple included studies report higher IL-1β and IL-6 among CHIP carriers,^9,13^ dovetailing with the observation that patients with *TET2*-CHIP may obtain greater clinical benefit from IL-1β blockade with canakinumab in exploratory analyses of CANTOS.^25^ While the CHIP–canakinumab interaction was hypothesis generating, it is consistent with preclinical data and suggests that anti-inflammatory strategies might be enriched in post-ACS patients with specific CHIP genotypes. Notably, broader anti-inflammatory programmes (e.g. IL-6 receptor blockade in ASSAIL-MI) have shown biomarker and salvage benefits without clear impacts on hard endpoints, highlighting that patient selection, potentially informed by CHIP status, may be crucial.^26,27^

Metabolic context may influence CHIP-associated risk, although evidence for formal effect modification remains limited. Zhao *et al*. reported a stronger adverse association among patients with T2DM and presented bidirectional Mendelian randomization consistent with metabolic–inflammatory interaction.^12^ This is concordant with experimental observations that *Tet2*-mutant CH aggravates metabolic dysfunction,^28^ and with clinical signals of higher NT-proBNP and inflammatory cytokines in carriers, parameters that are closely related to adverse ventricular remodelling. However, although individual cohorts have explored metabolic context, the present meta-analysis was underpowered to formally assess association modification by comorbidity.

Nonetheless, exploratory analyses suggested that study-level differences in smoking prevalence between CHIP carriers and non-carriers were associated with variability in association estimates, with a tendency towards stronger CHIP–mortality associations in cohorts where smoking prevalence was lower among carriers. This pattern is consistent with a potential negative confounding or association-modifying role of smoking, whereby the pro-inflammatory and pro-atherothrombotic consequences of CHIP may be more apparent in the absence of competing inflammatory exposures such as tobacco use. Importantly, these observations are based on aggregate, study-level data and are exploratory and hypothesis-generating rather than evidence of a causal interaction.

Despite these findings, these studies are limited by substantial clinical and methodological heterogeneity. Sampling windows varied, which can alter apparent VAF through peri-PCI haemodilution/leucocytosis or survivor bias. Assays ranged from ∼200× whole-exome to ultra-deep UMI-based panels (>10 000×), influencing sensitivity for small clones, apparent CHIP prevalence, and interpretation of harmonized VAF thresholds. Variation in gene coverage (two-gene vs broad panels) further complicates comparisons. Endpoints spanned 30-day death/RRT in shock to 10-year MACE/MACCE, with inconsistent inclusion of HF components. Some adjusted models incorporated IL-6 or NT-proBNP, which are possible mediators, and likely to bias estimates towards the null. Finally, most clinical datasets were underpowered for modest between-gene differences. For example, *JAK2* mutations were rare and estimates imprecise, although its pro-thrombotic biology may be relevant in MI.

Taken together, our study refines prior assumptions in three important ways. First, the cardiogenic-shock signal indicates that the adverse prognostic association of CHIP is not confined to chronic atherogenesis but extends into hyper-acute MI phases characterized by intense systemic inflammation and multiorgan stress. Second, across the available cohorts, we observed no consistent or reproducible differences in risk between individual driver mutations, with *DNMT3A*, *TET2*, and *ASXL1* showing broadly comparable associations with adverse outcomes where assessed. That said, several studies reported numerically stronger associations for *TET2*; however, these signals were not uniform across cohorts, and we did not have sufficient power to make mutation-specific inferences. Third, the magnitude of reported associations varied substantially between studies, possibly reflecting heterogeneity in clinical context, sequencing methodology, exposure definition, and outcome ascertainment. More broadly, CHIP may represent one component of systemic ageing biology relevant to post-ACS ventricular dysfunction and HF, alongside inflammatory, metabolic, and cellular senescence pathways described in contemporary HF frameworks.^29^

Looking forward, the field needs (i) adequately powered, standardized prospective cohorts with uniform sampling windows and ultra-deep panels to enable gene-specific and clone-size risk estimation; (ii) systematic capture of HF outcomes (left ventricular volumes/strain, NT-proBNP, HF hospitalization); (iii) mechanistic studies that map specific mutations to discrete inflammatory, thrombotic, and fibrotic pathways; and (iv) genotype-enriched interventional trials to move towards personalized therapy.

### Limitations

This analysis has several limitations. All included studies were observational, and residual confounding cannot be excluded. Furthermore, in some cohorts, adjustment included inflammatory biomarkers that may be causally related to the observed associations. Clonal haematopoiesis of indeterminate potential ascertainment varied widely with respect to sequencing depth, gene coverage, VAF thresholds, and sampling timing, limiting comparability across studies and constraining inference regarding clone-size and mutation-specific associations. Although prespecified sensitivity analyses were used to address potential cohort overlap, incomplete exclusion of non-independence, particularly within large national STEMI programmes, remains possible. Power was limited for mutation-specific and comorbidity-stratified analyses, which should therefore be considered hypothesis generating. Finally, outcome definitions varied across studies, and reliance on all-cause mortality, while maximizing comparability, may obscure cause-specific mechanisms. Accordingly, the absence of detected statistical heterogeneity should not be taken as evidence that the included cohorts were clinically or methodologically homogeneous.

## Conclusions

In this systematic review and meta-analysis, CHIP was associated with increased all-cause mortality following ACS, spanning low-risk ACS, unselected STEMI populations, and cardiogenic shock. Future research should focus on prospective, standardized studies capable of resolving gene- and clone-size–specific risks, clarifying interactions with clinical variables, systematically capturing ventricular dysfunction and HF outcomes, and determining whether targeted anti-inflammatory or genotype-enriched strategies can improve outcomes.

## Supplementary Material

xvag191_Supplementary_Data

## Declarations

### Data Availability

No data were generated or analysed for this manuscript.

### Pre-registered Clinical Trial Number

None supplied.
