# Supplementary material for: Clonal haematopoiesis and outcomes after acute coronary syndrome: a systematic review and meta-analysis
Source: ESC Heart Fail. 2026 Jul 6;13(5):xvag191. doi: 10.1093/eschf/xvag191 (PMC13695070; doi:10.1093/eschf/xvag191)
Supplement: Supplementary file 1 [file xvag191_supplementary_data.docx]

clonal haematopoiesis and outcomes after acute coronary syndrome: a systematic review and meta-analysis

Supplementary Material

Supplementary methods

Search strategy

1. Exp myocardial infarction/
2. (Anterior wall myocardial infarction or AMI or inferior wall myocardial infarction or acute myocardial infarction or non-ST-elevation myocardial infarction or NSTEMI or ST-elevation myocardial infarction or STEMI)
3. 1 or 2
4. Exp clonal haematopoiesis/
5. (Clonal haematopoiesis or Clonal haematopoiesis)
6. 4 or 5
7. 3 and 6
8. 7 not exp “Review”/
9. Limit 8 to human
10. Limit 9 to English language

Supplementary Tables

**Supplementary Table 1.** Database-specific controlled vocabulary terms used in the search strategy.

| **Search term** | **Database** | |
| --- | --- | --- |
|  | **Embase** | **Medline** |
| Myocardial Infarction | heart infarction (Emtree) | Myocardial infarction (MeSH) |
| Anterior Wall Myocardial Infarction | Anterior myocardial infarction (Emtree) | Anterior Wall Myocardial Infarction (MeSH) |
| AMI | Acute heart infarction (Emtree) | Myocardial Infarction (MeSH) |
| Inferior Wall Myocardial infarction | Inferior myocardial infarction (Emtree) | Inferior Wall Myocardial Infarction (MeSH) |
| Acute myocardial infarction | Acute heart infarction (Emtree) | Myocardial Infarction (MeSH) |
| Non-ST Elevated Myocardial Infarction | non ST segment elevation myocardial infarction (Emtree) | Non-ST Elevated Myocardial Infarction (MeSH) |
| NSTEMI | non ST segment elevation myocardial infarction (Emtree) | Non-ST Elevated Myocardial Infarction (MeSH) |
| ST Elevation Myocardial Infarction | ST segment elevation myocardial infarction (Emtree) | ST Elevation Myocardial Infarction (MeSH) |
| STEMI | ST segment elevation myocardial infarction (Emtree) | ST Elevation Myocardial Infarction (MeSH) |
| Clonal Hematopoiesis | clonal hematopoiesis (Emtree) | Clonal Hematopoiesis (MeSH) |
| Clonal Haematopoiesis | clonal hematopoiesis (Emtree) | Clonal Hematopoiesis (MeSH) |

ACS, acute coronary syndrome; AMI, acute myocardial infarction; NSTEMI, non-ST-elevation myocardial infarction; STEMI, ST-elevation myocardial infarction.

**Supplementary Table 2.** Covariates included in adjusted models used for quantitative synthesis.

| **Study** | **Effect estimate used in quantitative synthesis** | **Covariates included in adjusted model** | **Notes** |
| --- | --- | --- | --- |
| Wang et al.(1) | Adjusted time-to-event estimate for all-cause mortality | Multivariable model including demographic characteristics, cardiovascular risk factors, laboratory variables, left ventricular ejection fraction, renal function, and treatment variables where reported | Prospective STEMI cohort; DNMT3A/TET2-focused analysis. |
| Jiang et al.(2) | Age-adjusted time-to-event estimate for all-cause mortality/MACCE where reported | Age | Stringent cardiovascular risk factor exclusions limited conventional covariate adjustment. |
| Böhme et al.(3) | Adjusted estimate for all-cause mortality or death/RRT composite | Age, sex, randomised PCI strategy, and CLIP score | CLIP score includes cystatin C, lactate, interleukin-6, and NT-proBNP; possible mediator adjustment considered in interpretation. |
| Zhao et al.(4) | Adjusted time-to-event estimate for all-cause mortality | Multivariable model including demographic characteristics, cardiovascular risk factors, metabolic variables, renal function, infarct/procedural characteristics, and treatment variables where reported | Fuwai/CRGAM-derived cohort; diabetes-stratified analysis. |
| Li et al.(5) | Adjusted time-to-event estimate for all-cause mortality/clinical events | Multivariable model including demographic characteristics, cardiovascular risk factors, laboratory variables, infarct/procedural characteristics, and treatment variables where reported | Fuwai/CRGAM-derived cohort; imaging-enriched analysis. |
| Fan et al.(6) | Adjusted time-to-event estimate for all-cause mortality/MACE | Multivariable model including demographic characteristics and reported clinical covariates | Day-28 sampling; potential survivor bias considered in interpretation. |
| Zhao et al.(7) | Adjusted time-to-event estimate for all-cause mortality | Multivariable model including demographic characteristics, cardiovascular risk factors, lipid/metabolic variables including atherogenic index of plasma, renal function, infarct/procedural characteristics, and treatment variables where reported | Fuwai/CRGAM-derived cohort; treated as potentially overlapping. |
| Sun et al.(8) | Adjusted time-to-event estimate for all-cause mortality/MACE | Multivariable model including demographic characteristics, cardiovascular risk factors, laboratory variables, infarct/procedural characteristics, and OCT-derived plaque characteristics where reported | Harbin Medical University cohort; possible overlap with Wang et al. considered in sensitivity analyses. |

Supplementary Figures

**Supplementary Fig 1. Sensitivity analysis excluding potentially overlapping cohorts.** Forest plot showing pooled adjusted associations between clonal haematopoiesis of indeterminate potential (CHIP) and all-cause mortality after acute coronary syndrome (ACS), after exclusion of studies with possible cohort overlap.

**Supplementary Fig 2. Sensitivity analysis excluding cohorts with only unadjusted estimates.** Forest plot showing pooled adjusted associations between clonal haematopoiesis of indeterminate potential (CHIP) and all-cause mortality after acute coronary syndrome (ACS) after exclusion of studies including only unadjusted estimates.

**Supplementary Fig 3. Influence analysis.** Leave-one-out analysis showing the effect of sequentially excluding individual studies on the pooled estimate for all-cause mortality.

**Supplementary Fig 4. Influence analysis.** Leave-one-out analysis showing the effect of sequentially excluding individual studies on the pooled estimate in sensitivity analyses excluding A) studies with possible cohort overlap; and B) studies including only unadjusted estimates.

**Supplementary Fig 5. Influence analysis.** Leave-one-out analysis showing the effect of sequentially excluding individual studies on the pooled estimate: A) *ASXL1*; B) *TET2*; and C) *DNMT3A*. These analyses are limited by the small number of contributing studies per subgroup.

Supplementary References

1.	Wang S, Hu S, Luo X, Bao X, Li J, Liu M, et al. Prevalence and prognostic significance of DNMT3A- and TET2- clonal haematopoiesis-driver mutations in patients presenting with ST-segment elevation myocardial infarction. EBioMedicine. 2022;78:103964.

2.	Jiang Z, Li Y, Yan C, Zhang X, Zhang Q, Li J, et al. Clonal hematopoiesis of indeterminate potential in patients with acute coronary syndrome undergoing percutaneous coronary intervention in the absence of traditional risk factors. Clin Res Cardiol. 2023;112(4):506-17.

3.	Bohme M, Desch S, Rosolowski M, Scholz M, Krohn K, Buttner P, et al. Impact of Clonal Hematopoiesis in Patients With Cardiogenic Shock Complicating Acute Myocardial Infarction. J Am Coll Cardiol. 2022;80(16):1545-56.

4.	Zhao X, Li J, Yan S, Tan Y, Chen R, Li N, et al. Clonal hematopoiesis of indeterminate potential and type 2 diabetes mellitus among patients with STEMI: from a prospective cohort study combing bidirectional Mendelian randomization. Cardiovasc Diabetol. 2025;24(1):28.

5.	Li J, Chen R, Zhou J, Li N, Zhou P, Liu C, et al. Effect of clonal hematopoiesis on plaque morphology and prognosis in patients with acute myocardial infarction. Genome Med. 2025;17(1):94.

6.	Fan WL, Yeh JK, Hsieh LC, Tsai ML, Ho MY, Huang YC, et al. Prognostic significance of clonal hematopoiesis in STEMI: a 10-year follow-up reveals high-risk gene mutations. Hum Genomics. 2025;19(1):51.

7.	Zhao X, Li J, Chen R, Li N, Xue L, Yan S, et al. The predictive value of atherogenic index of plasma and clonal hematopoiesis of indeterminate potential among patients with STEMI-from a prospective cohort study. Cardiovasc Diabetol. 2025;24(1):333.

8.	Sun Q, Wang S, Zeng M, Liu M, Zhao C, Yi B, et al. Impact of Carrying DNMT3A or TET2 Mutations on Plaque Characteristics and Prognosis in Patients With STEMI Based on OCT. Circ Cardiovasc Imaging. 2025;18(9):e017915.
